# Research Capacity Strengthening in American Samoa: Fa’avaeina le Fa’atelega o le Tomai Sa’ili’ili i Amerika Samoa

**DOI:** 10.1093/bjsw/bcz160

**Published:** 2019-12-31

**Authors:** Va’atausili Tofaeono, Lana Sue I Ka’opua, Angela Sy, Tyran Terada, Rachelann Taliloa-Vai Purcell, Salote Aoelua-Fanene, Katherine Tong, Victor Tofaeono, Tofoipupu Unutoa-Mageo, Luana Scanlan, Kevin Cassel, Adelaida Rosario

**Affiliations:** 1 American Samoa Community Cancer Coalition, Pago Pago, AS, USA; 2 Cancer Center, University of Hawaii, Honolulu, HI, USA; 3 Myron B Thompson School of Social Work, University of Hawaii, Manoa, Honolulu, HI, USA; 4 John A Burns School of Medicine, University of Hawaii, Manoa, Honolulu, HI, USA; 5 Department of Nursing, Vanguard University, Costa Mesa, CA, USA; 6 United States Public Health Service, Denver, CO, USA; 7 National Institute of Minority Health and Health Disparities, Bethesda, MD, USA

**Keywords:** American Samoa, culture, health disparities, research capacity, social determinants of health, US territories

## Abstract

Capacity-building partnerships are central to the sustainable development goals (SDGs), the UN’s blueprint for achieving global health equity. The UN Permanent Forum on Indigenous Issues endorses the SDG and underscores the need for global partnerships that respect local leadership and culture. Innovations that weave or integrate Indigenous and Western knowledges are emphasised. These recommendations guided the INdigenous Samoan Partnership to Initiate Research Excellence (INSPIRE). INSPIRE is led by investigators from American Samoa and supported by US co-investigators. In project year one, INSPIRE queried: What weaving approaches are feasible for promoting community access to INSPIRE’s research hub and for training Indigenous researchers? Weaving procedures involved interlacing Samoan and Western knowledges. Cultural tailoring strategies were used to customise communications. Formative evaluation suggests the feasibility of INSPIRE’s efforts. Evidential tailoring provided information on American Samoa (A.S.) social determinants of health; trainees indicated increased research commitment. Linguistic and sociocultural relevance tailoring were positively received; trainees reported increased interest in research praxis and initiated an A.S. research capacity-strengthening model. Social work assured knowledge parity in development/delivery of the training curriculum and culturally safe discussions on social determinants of health, territorial status and Samoan survivance. Findings are context-specific yet offer considerations for capacity-strengthening partnerships seeking to advance health equity.

## Background and significance

### Global health equity, the call to leave no one behind and reach the furthest first

The World Health Organization (WHO, 2018) states unequivocally that disparate health outcomes occurring between and within countries are influenced by pernicious social determinants of health (SDOH)—the socially constructed economic, political and sociocultural factors that negatively influence human longevity and a community’s quality of life. In 2000, the UN adopted the Millennium Development Goals (MDGs), with the vision of ending poverty and advancing health equity. However, post-2015 reports indicated that MDG advancement was uneven and continuing efforts were needed to increase progress in developing nation states and among historically marginalised Indigenous people (United Nations Department of Economic and Social Affairs, 2014, 2018). Critics noted that partnerships between developing and developed nation states often reflected ‘the West lectures the rest’ dynamic, as characterised by Western knowledge hegemony and intervention programmes designed by outside experts (Schuftan, 2015). This dynamic disempowered developing nation states, compromised intervention relevance and inhibited sustainable development. Post-2015 MDG assessment led to *Transforming Our World: The 2030 Agenda for Sustainable Development* report and articulation of seventeen sustainable development goals (SDGs). SDG #17 focuses on empowering global partnerships and is regarded as the key to unlocking all other goals (United Nations Division for Sustainable Development, 2018). The SDG’s overall call to action is ‘Leave no one behind and reach the furthest first.’

Indigenous people have protracted histories of being left behind. The UN Permanent Forum on Indigenous Issues and the Small Island Developing States (SIDS) collective endorse the SDG, while at the same time, advocating for partnerships that foster parity of Indigenous and Western knowledges with attention to cultural lifeways, respect for local leadership and animation of opportunities for meaningful community participation (United Nations Department of Economic and Social Affairs, 2014, 2018). ‘Weaving’ Indigenous and Western knowledges are understood to advance health equity for the Indigenous community whose own knowledge and values are often subsumed (Cultural Survival, 2018).

Indigenous people living in SIDS like the US territories are disproportionately burdened by poverty and health disparities (Cottle, 1995; US Census Bureau Citizens’ report: FY, 2010; Ka‘opua *et al.*, 2011a, b; Palafox and Hixon, 2011; Rodriguez-Vila *et al.*, 2014). This article describes the process of ‘fa’avaeina le fa’atelega o le tomai saili’ili i Amerika Samoa’ (‘establishing a foundation for research capacity strengthening in American Samoa’), which occurred in Year one of a five-year project. The INdigenous Samoan Partnership to Initiate Research Excellence (INSPIRE) is a research capacity-strengthening programme supported by a competitive grant from the National Institute of Minority Health and Health Disparities. Health research capacity building is INSPIRE’s overall goal. Colorectal cancer (CRC) screening provides the ‘case’ or specific learning medium for health disparities research training.

CRC screening was selected as the learning medium because it is a priority of the US Department of Health and Human Services, as well as of the US Affiliated Pacific Islands Cancer Coalition (Palafox and Hixon, 2011; American Samoa Community Cancer Coalition, 2013; US Department of Health and Human Services, Center for Medicare and Medicaid Services (CMS), 2016). From 2008 to 2013, the percentage of US adults, aged 50–75 years who participated in CRC screening, was about 58 per cent and evidenced a steady advance towards achieving the Health People 2020 target of 70 per cent participation (Health and Human Services, US Preventative Task Force, 2015). By comparison, the American Samoa (hereafter, known as Amerika Samoa or A.S. except when referencing a proper noun or territorial-specific matter) Behavioural Risk Factor Surveillance System data indicate that less than 7 per cent of age-eligible A.S. adults participated in CRC screening, even when screening was available (American Samoa Department of Health, 2013; American Samoa Government, 2018).

INSPIRE’s principal investigators are Indigenous Samoans and Pacific Islanders residing in the tradition-rich, yet resource-constrained Territory of A.S., US co-investigators are affiliated with US Research Tier 1 and M3 universities. The partnership includes individuals trained in business, medicine, nursing, public health and social work. In co-developing the proposal that funds INSPIRE, partners agreed that: (i) A.S. investigators are the ultimate arbiters of all project decisions; (ii) programme activities will promote knowledge parity by lalaga (weaving) Samoan and Western ways of knowing; and (iii) that these agreements will undergird research applications.

In project year one, INSPIRE’s specific aims were to: (i) develop/deliver a research training curriculum and (ii) establish/outfit a community-accessible research hub. INSPIRE’s research questions were: What weaving approaches are feasible for training Indigenous researchers? For promoting community access to INSPIRE’s research hub?

The American Samoa Government (ASG) Department of Health (DOH) institutional review board awarded ethical approval for this research. Persons receiving the pilot research curriculum (trainees) provided written informed consent.

### History of colonisation, US territorial status, cultural survivance

Indigenous Samoans are the oceanic people who settled the Samoan Archipelago nearly 3,000 years ago (American Samoa Historic Preservation Office, 2018). Prior to European and American contact, an Indigenous governance system known as fa’amatai (way of the chiefs) was organised. This system was ensured through practice of fa’asamoa (Samoan ways), lifeways rich in collectivist values that guided cooperative work and learning translated as 'E a le puga nisi, a le ‘ana nisi' (let each do their share of the work) (ASG, American Samoa's Comprehensive Economic Development Strategy, 2006). Alaga‘upu (ancestral wisdoms) stressed responsibility to share in the work deemed essential for the well-being of all and are used in contemporary times (One Samoana, 2018).

Prior to the advent of Western colonisation, Indigenous science flourished and produced innovations in astronomy, oceanic navigation and long-distance canoe-building (Polynesian Voyaging Society, 2010). Historic migratory voyages exemplify success in weaving collectivist tradition with scientific innovation. High-precision radiocarbon dating of material culture and proto-linguistic analyses of the twenty-first century indicates that the ancient peoples from the region in and around the Samoan Archipelago spawned initial migrations resulting in settlement of the Polynesian Triangle—the area bounded by Hawai‘i, Rapa Nui (Easter Island) and Aotearoa (New Zealand). Thus, the region in and around the Samoan Archipelago is esteemed as the Polynesian Cradle—the place where Polynesian tradition and innovation originate.

The US Navy entered the Polynesian Cradle in 1827 (US Department of the Interior [DOI], Office of Insular Affairs [OIA], 2018). Enabled by the rhetoric of Manifest Destiny and the Whiteman’s Burden, the USA justified its moral imperative to expand influence beyond the North American continent, thereby competing with European nations for control of the Samoan Archipelago and other Indigenous lands (Correia, 2009). In the Tripartite Treaty of 1899, the USA together with Great Britain and Germany, partitioned the Samoan Archipelago, allegedly to end hostilities between Samoan clans (US Department of the Interior [DOI], Office of Insular Affairs [OIA], 2018). The USA assumed sovereignty of the eastern archipelago, with deeds of cession executed in 1929, thereby creating the Territory of American Samoa. Under US constitutional law, the USA holds plenary power (absolute authority without review or limitations in exercising that power), with territorial oversight that currently resides with the Department of the Interior Office of Insular Affairs (DOI-OIA). Territorial status is associated with significant and pernicious social determinants of health, detailed in the later section.

Although challenged by harmful, distal SDOH, A.S. remains rich in its Indigenous culture. Cultural survivance is actively practiced and passed on across generations (American Samoa Government, 2018; Vizenor, 2008). The current A.S. population is estimated at 57,798, with nearly 90 per cent of all residents identifying as ethnic Samoan (Country Meters, 2018). ‘Samoa muamua le Atua’ (‘Let God be first’) is the motto of A.S., with about 98 per cent of ethnic Samoans indicating that they are Christian (American Samoa Government, 2018). Samoan and English are the official languages and a high percentage of the population is bi-lingual. Culture is understood to be carried through language; thus, gagana Samoa (Samoan language) is consciously perpetuated. An estimated 91 per cent of the ethnic Samoan population use their mother tongue at home. Both English and Samoan languages are used in the workplace, with Samoan often used to convey contextual meanings that are not adequately expressed in English (Ka‘opua *et al.*, 2010; American Samoa Government, 2018).

### SDOH, health disparities, US territories, Amerika Samoa

The US territories are considered SIDS by the UN and are understood to face unique challenges in achieving health outcomes articulated in the SDGs (United Nations Department of Economic and Social Affairs, 2014). ‘Federal policy issues concerning the territories are decided in the absence of any guiding federal policy or consideration of the effects on those territories. These islands remain in a limbo neither foreign nor domestic…a condition that complicates development of solutions’ (Tulafono, 2012). In comparison to the US states, the territories have significantly higher levels of poverty and are disproportionately encumbered by cancer and other health disparities (US Census Bureau Citizens’ report: FY, 2010; US DOI-OIA, 2018). Critics posit that territorial status is a distal SDOH operating through political and other factors (Stayman, 2009).

#### Political challenges

A.S. and the four other US territories (Guam, Commonwealth of the Northern Mariana Islands, Puerto Rico, Virgin Islands) are subject to US constitutional law, plenary power, federal laws and treaties; however, the territories remain unable to influence policies that directly affect their health and well-being (Cottle, 1995; Stayman, 2009; Morrison, 2013; Rodriguez-Vila *et al.*, 2014). Under US constitutional law, territorial residents are without suffrage in US presidential elections and their Congressional representatives have no vote. Notably, A.S. residents are unique in that they are not accorded as US birth right citizenship and are designated as US nationals. As such, Samoans carry an A.S. passport and if they choose, they may apply for US citizenship.

#### Socioeconomic challenges

Poverty is the most robust SDOH, with ill health presenting a cascade of barriers (WHO, 2018). A.S. health is influenced by low socioeconomic status at several critical levels. First, a country or region’s Gross Domestic Product assesses an area’s economic health and gauges standard of living. At the territorial level, a DOI-OIA-commissioned report found that A.S. net imports are greater than exports for every year from 2002 to 2009 thus signalling an economy in trouble (Osman, 2012). Grant assistance may be helpful in coping with this shortfall, but insufficient without specific efforts for economic revitalisation and attention to the impact of US policies and trade agreements on A.S. Secondly, the overall US poverty rate by household income with geographic adjustment is about 14.8 per cent (US Census Bureau Citizens’ report: FY, 2010). By comparison, the A.S. poverty rate is estimated at 57.8 per cent. Notably, this poverty rate is higher than that of all US states, the District of Columbia and all other US territories (DOI-OIA, 2018; US Census Bureau Citizens’ report: FY, 2010). Because a high percentage of Indigenous Samoans live at or below the US Federal poverty level, Medicaid funds are directed to the territory’s sole medical entity, which provides health care services with minimal patient co-payment. (US Department of Health and Human Services, Center for Medicare and Medicaid Services, 2016). Thirdly, A.S. is expected to contribute a non-federal share to access Medicare and Medicaid funds. This has been a persistent challenge to a territorial economy with a negative trade balance and struggling to survive (Stayman, 2009).

#### Data challenges

US territories are eligible to apply for most US funding opportunities. However, resource-constrained territorial entities are hindered by the lack of funds to develop and sustain a research infrastructure (Ka‘opua *et al.*, 2011a). Inadequate health and other data place the five US territories at profound disadvantage when competing against US states and US-dwelling Indigenous peoples (Alaska Natives, Native Americans, Native Hawaiians) who have access to more developed research infrastructures. Frequently, territorial statistics are not collected or if collected are analysed as an aggregate with the US states thereby, obscuring A.S. needs (US Department of the Interior [DOI], Office of Insular Affairs [OIA], 2008). Furthermore, territorial health research is limited by an epidemiological transition in which scarce funds must be used to address both communicable and non-communicable diseases.

The unavailability of current and reliable data on A.S. residents, lack of research infrastructure and cancer and other health disparities are of significant concern. Indicated is the need for partnerships focussed on producing A.S.-specific research and strengthening Indigenous Samoan research capacity.

### Research capacity strengthening, Pacific Islander scholarship, de-colonising research

Research capacity strengthening (RCS) is the evidence-informed process of increasing knowledge, attitudes and skills requisite to conducting rigorous inquiry on issues identified as relevant by community stakeholders; this seems especially vital for resource-constrained, yet culturally rich communities (Essence on Health Research, 2014; Sitthi-amorn and Somrongthong, 2000). RCS emphasises attention to sustaining capacity at several levels. At the individual level, RCS efforts are leveraged by training on research knowledge and skill development, with opportunities to participate in scientific activities. At the organisational level, RCS focuses on developing interventions that align with community needs and cultural lifeways. Support of local leadership and community ownership are crucial to ensure research relevance and sustainable development (Essence on Health Research, 2014).

Pacific Islander scholarship underscores de-colonisation of research and promotion of parity between Indigenous and Western knowledges. De-colonisation of research necessarily involves culturally safe and tailored research praxis. Such praxis is premised on the strengths-based perspective and demonstrates appreciation for reciprocal learning, monitoring of negative biases and resisting cultural evangelism—a variation of ‘the West lectures the rest’ dynamic (Ka‘opua *et al.*, 2014, 2017; Schuftan, 2015).

Samoan oral tradition uses alaga’upu (ancestral wisdoms) usually expressed through culturally grounded metaphors. Published Samoan scholarship follows this tradition. Metaphors such as lalaga or tui (weaving), fale (house, place for dwelling, meeting, weaving) and va’a (canoe) have been used to advance culturally relevant, health/mental health research and practice. Pulotu-Endemann (2001) is credited with the seminal ‘weaving’ model; the Falefono Model features parts of the Samoan dwelling to describe and organise values relevant to sexual health promotion. Tamasese and colleagues (2005) and Seiuli (2012) use fale and tui/lalaga metaphors to advance Indigenous Samoan research methods. Notably, two social work models are articulated. Mulitalo-Lauta (2000) enunciates the Lalaga (weaving) Model that integrates Samoan cultural values and social work practice within the Aoteaora/New Zealand context. Oceanic voyaging is the overarching metaphor used in Tauta’i Lavea’i, an A.S. breast cancer patient navigation pilot study (Ka‘opua *et al.*, 2010). ‘Tauta’i’ refers to ‘navigating’ a canoe as in trans-Pacific voyaging; ‘lavea’i’ refers to ‘protection from harm’. Weaving such metaphors into the breast cancer patient navigation programme enhanced acceptability and facilitated intervention uptake. The overall body of published Samoan scholarship informs INSPIRE’s methods for knowledge weaving and culturally tailored communications.

## Method

Typically, researchers assess feasibility prior to conducting an efficacy intervention trial. Feasibility assessment provides preliminary evidence that an innovation is acceptable to the group for which it is intended (Ka‘opua *et al.*, 2011b, 2014). In project year one, INSPIRE’s aims were to: (i) develop and deliver a health research curriculum relevant to the A.S. context and (ii) establish an interactive and accessible research hub. Research questions focused on weaving approaches feasible for promoting community use of INSPIRE’s research hub and for training Indigenous Samoan researchers.

### Procedures

Weaving and cultural tailoring were key to intervention development and involved discussion among partners, as well as consultation with INSPIRE’s advisory board and other community members. Weaving procedures focused on interlacing culturally diverse, yet potentially complementary Samoan and Western perspectives and practices. Cultural tailoring procedures focused on communication messages and activities. Upon clarifying ‘who’ INSPIRE sought to influence (i.e. research trainees, A.S. health and research organisations, community leaders, general community), INSPIRE activities and communications were tailored using peripheral, evidential, linguistic, constituent-involving and relevant sociocultural strategies (Ka‘opua *et al.*, 2011b, 2014).

#### Aim #1. Le va fealoalo’ai, the hub for research resources and interaction

INSPIRE situated le va fealoalo’ai in an office suite replete with research-relevant resources, including rooms for meetings and conferences, cubicles for individual work, computer terminals, high-speed internet access, software for quantitative and qualitative data management and analyses, audio-visual capability, learning platforms for distance education, educational print materials and software for quantitiative data collection. These standard scholastic resources were previously not available to all in resource-constrained A.S, yet are basic and essential to RCS.

From the Samoan cultural perspective, knowledge-building is viewed as a collective process with sharing of resources; as such, products optimally are owned and available to all (Lesa, 1981; Tamasese *et al.*, 2005). Thus, le va was designed to be accessible to all. Le va was established in a commercial area frequented by residents and accessible by public transportation. The physical space was designed to be welcoming. Le va was peripherally tailored for cultural acceptability. Photographs of INSPIRE partners and community members involved in cancer health-related activities are displayed in INSPIRE’s lobby and meeting rooms. Project print materials are readily available and carried the INSPIRE logo. The logo features cultural symbols (e.g. tatau or traditional tattoos) and is an example of peripheral (visual) tailoring.

An open house reception was held to encourage community access and ownership. The reception commenced with a traditional ‘ava ceremony. ‘Ava is a beverage made throughout Polynesia and the ‘ava ceremony is a centuries-old, honouring ritual (Grattan, 1985). INSPIRE’s ‘ava ceremony involved traditional, religious and political leaders, orators, le taupo (chief’s daughter responsible for beverage preparation) and ‘aumaga (untitled young men designated as caretakers and helpers in serving the ‘ava). INSPIRE’s ‘ava ceremony was followed by a shared meal and project-related presentations that conveyed the weaving of Indigenous Samoan and Western ways of knowing. The sum of these activities reflected visual and oral use of communications tailored on peripheral, evidential, linguistically appropriate, constituent-involving and socioculturally relevant elements.

#### Aim #2. Tomai sa’ili’ili (knowledge-seeking), development/delivery of research curriculum

The inaugural training cohort included persons who voluntarily participated: seven individuals completed the training (four master’s-trained, three doctorate-trained). Praxis intervention, a form of participatory action research, undergirded course units delivered over a period of 15 weeks (Ford and Airhihenbuwa, 2010). The weaving of Indigenous Samoan and Western knowledges was operationalised by use of research textbook from the USA (Friedman, 2017), as well as published Indigenous health scholarship and global, USA and A.S. health agency reports. Reports were selected for relevance to A.S., cancer disparities, SDOH and community-based participatory, action-oriented research. Instructional strategies followed principles of Participatory Education and contemporary Samoan pedagogy (Lesa, 1981; Campbell and Burnaby, 2001; Ka‘opua *et al.*, 2011b, 2014; Aoelua-Fanene, 2017). Alaga’upu (Samoan wisdoms) were used to incentivise and reinforce positive ways of work. Faculty with background in cultural tailoring of health education and community-based participatory research provided training. Faculty facilitated critically analytic discussion of Western and Indigenous scholarship, with implications for A.S. research praxis. Distance technologies allowed partners in the US states to provide research instruction and facilitate/participate in discussions.

## Findings

### 

Trainees preferred to work together on most major deliverables (e.g. articulation of research questions, design of a community health intervention, coding qualitative data). Trainees discussed their learning after each session. Upon completion of all units, trainees completed an anonymous course evaluation. The evaluation included items with rating scales, as well as open-ended questions. Table 1 highlights INSPIRE’s use of cultural tailoring strategies, weaving examples and suggested outcomes.

**Table 1:** Strategies for cultural tailoring, weaving examples, suggested outcomes

Strategies for cultural tailoring	Feasible approaches, examples, suggested outcomes
Peripheral Definition: Visual images used to enhance appeal and support message Desired result: Person believes that proposed action will benefit health and well-being	INSPIRE logo. Print materials feature project logo, which uses visual images that reflect Samoan culture. Photographs. Displayed in research hub, meeting room and training presentations are photographs of Samoan community members taking health-related actions. Suggested outcome: In Year 1, 170 persons accessed the INSPIRE hub of their own volition and ∼75 per cent returned for additional contact
Evidential Definition: Print materials and presentations provide facts and information Desired result: Person believes that the health-related issue is important and action-oriented behaviour is indicated.	INSPIRE curriculum: A research textbook was used and augmented by published readings on Indigenous science and research methods. Attention given to inclusion of Indigenous scholarship that ‘weaves’ Western and Samoan knowledges. INSPIRE activities: Trainees analysed and discussed reading materials and lecture presentations on CRC screening, factors related to current disparities and policy statements. Suggested outcome: Evidential and socioculturally relevant materials were endorsed for use in future training. Trainees reported increased commitment to screening initiatives and found materials relevant to research praxis.
Linguistic Definition: Words, phrases and terms common to Indigenous language are purposefully used, with attention to cultural norms, values and layered meanings. Desired result: Use of familiar terms clarifies and supports learning gleaned through reading, presentations and learning activities.	Purposeful use of Samoan language. Alaga’upu (ancestral wisdoms) used to encourage desirable ways of working together (e.g. ‘Ia su’i tonu le mata o le nui’/’There is a proper way of undertaking tasks’; ‘O le ala i le pule o le tautua’/ ‘The pathway to leadership is through service to others’). Bi-lingual communications. Samoan and English languages used in INSPIRE communications with trainees and community. Suggested outcome: Trainees reported enthusiasm for future activities, including development of health literacy and Knowledge-Attitudes-Practice measures in Samoan and English languages.
Constituent-involving Definition: Learning draws upon collective experiences. Desired result: Persons involved have buy-in and receptive to facilitating multi-sectorial involvement.	Community guidance. INSPIRE’s community advisory board routinely consulted. Broad promotion, with opportunities for meaningful involvement. INSPIRE avows/promotes local community use of INSPIRE resources, participation in community training and other activities. Suggested outcome: INSPIRE allows organisations and community groups to use conference facilities, thereby providing familiarity with the hub. This resulted in INSPIRE’s formal association with STEM steering committee and Territorial Epidemiological Outcomes Workgroup.
Sociocultural relevance Definition: Recognises, affirms and works from group values, beliefs, behaviours and social context. Desired result: Persons, groups, systems are motivated to action.	Traditional ‘ava ceremony conducted to introduce INSPIRE to A.S. community and welcome US partners. Fa’asamoa (Samoan ways) preferences collectivism; ‘ava ceremony reinforces the value of collective knowledge-building and -ownership. Capacity-building activities continuously iterate relevance/application to A.S., with attention to community participation and local leadership. Suggested outcome: Association with STEM and other groups extends INSPIRE’s reach to students thereby, potentiating sustainable research capacity.

#### Aim #1. Le va fealoalo’ai (the hub for research resources and interaction)

Project documentation indicates that 170 (unduplicated) adult or teenage persons accessed le va of their own volition, with about 75 per cent returning for additional contact. Visitors to le va included organisational groups. Notably, the Science, Technology, Engineering and Math (STEM) steering committee and Territorial Epidemiological Outcome Workgroup made official visits. These visits resulted in INSPIRE’s membership in both groups and subsequent opportunities to collaborate on cross-cutting research activities. Also, le va was the primary site for INSPIRE’s research training. In written evaluation and discussion, trainees expressed appreciation for le va, which they found accessible, welcoming and well-supplied with relevant resources.

#### Aim #2: Tomai sa’ili’ili (knowledge-seeking), development/delivery of research curriculum

Upon completion of all training units, trainees completed anonymous, written evaluations. Evaluations used Likert scales (1 = low satisfaction through 5 = high satisfaction). Trainees indicated high to moderately high levels of satisfaction with lecture presentations, readings and learning activities. Trainees endorsed use of group learning activities (*M *=* *4.71, *SD* = 0.49), increased their commitment to health disparities research (*M *=* *4.29, *SD* = 0.76) and would recommend the training to others (*M *=* *4.51, *SD* = 0.79). Open-ended comments clarified responses to survey items and provided additional insight into trainee learning:

‘Discussion allowed me to compare Western culture with Samoan culture, individualism *versus* collectivism.’

‘I learned how Samoan culture may not align with Western methods.’

‘I felt connected to the culture, language, and people of American Samoa.’

‘From my position of privilege…I feel that participation in INSPIRE has helped my people.’

‘INSPIRE helped me more effectively apply Samoan traditions and values to health issues.’

Completion of written evaluations prompted informal trainee discussions on weaving knowledges and on cultural tailoring of communications, with specific implications for RCS in the A.S. context. Trainees subsequently initiated development of a conceptual model for RCS. Through ensuing talatalaga/talanoaga (culturally patterned discussion/consensus), trainees and investigators agreed upon and articulated Le Fale o So’ofa’atasiga (The House for Research) conceptual model.

Le Fale o So’ofa’atasiga features Samoan values and metaphors described in course reading and evidence trainees’ knowledge and understanding of curriculum materials (Lesa, 1981; Tamasese *et al.*, 2005; Seiuli, 2012; Aoelua-Fanene, 2017). The model depicts le fale (the house) built on le fa’avae (the foundation) of: tomai saili’ili (knowledge-seeking, research), service leadership to aiga potopoto (community), fa’asamoa (Samoan ways, culture) and values of felagoglagoma’i (cooperation) and fa’asoa (sharing). Pou (pillars) of the house represent learning strategies, including va’ai (observing), fa’aloga (listening), talanoaina (discussing), fa’aali (using/applying knowledge). Le taualuga (roof) symbolises ola fa’aleagaga (life according to the Spirit, faith) that shelters le va fealoalo’ai (the space for interaction) and protects le va as a place of cultural safety where all knowledges are honoured and culturally grounded, health innovations are advanced. Le Fale o So’ofa’atasiga, the Samoan RCS model, is displayed in Figure 2.

**Figure 1:**
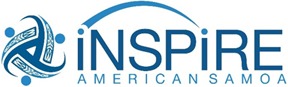
INSPIRE Logo: decided by the research cohort, the logo represents the collaboration between the partnering agencies: American Samoa Community Cancer Coalition, University of Hawaii, and Vanguard University (American Samoa Community Cancer Coalition, 2016).

**Figure 2:**
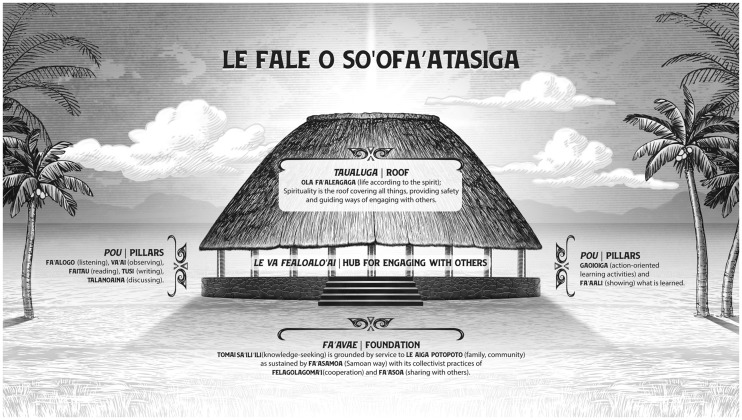
Le Fale o So’ofa’atasiga: Le fa’avae (foundation) is built on values of tomai saili’ili (knowledge-seeking, research), service leadership to aiga potopoto (community) and fa’asamoa (Samoan culture). As a collectivist culture, values of felagoglagoma’i (cooperation) and fa’asoa (sharing) are emphasised. Pou (pillars) of the house represent learning strategies, including va’ai (observing), fa’aloga (listening), talanoaina (discussing), fa’aali (using/applying knowledge). Le taualuga (roof) symbolises ola fa’aleagaga (life according to the Spirit, faith). Spirituality covers the place where Indigenous Samoan research is advanced. It ensures the cultural safety necessary for weaving all ways of knowing. Advances in culturally relevant health innovations thus are potentiated.

## Social work contributions

Ua gatasi le futia ma le umele.

While the fisherman swings the rod, the others must assist by paddling hard.

This alaga’upu (wisdom) uses fishing and paddling metaphors to exhort the value of diverse responsibility in the common endeavour*.* Social work/social welfare is a practice-based profession as well as an academic discipline. In research praxis, social work practice knowledge and skills converge with research knowledge and skills. In research praxis specific to INSPIRE, social work contributed: (i) perspectives on parity in development/delivery of the training curriculum and culturally safe; (ii) knowledge on strengths-based, ecological and culturally safe intervention; (iii) values of social and distributive justice; (iv) knowledge and skills in group process and practice with culturally grounded, resource-constrained communities; (v) use of participatory pedagogies promoting critical analyses; (vi) practical application of strategies for cultural-tailoring of INSPIRE’s research hub and training curriculum; and (vii) knowledge of CRC screening promotions—the learning medium for INSPIRE’s RCS efforts.

Notably, social workers co-facilitated highly sensitive, yet significant talatalaga/talanoaga (culturally patterned, focused discussion/consensus agreement) on territorial status, systemic discrimination and Samoan survivance. Such discussions involved ‘weaving’ supportive confrontation methods with talatalaga/talanoaga. Facilitation necessitated assurance of culturally safe praxis, with openness to diverse perspectives. This enabled mutuality of learning among discussants that reflected ‘leaving no one behind’ (United Nations Division for Sustainable Development, 2018), provided a positive alternative to ‘the West lectures the rest’ dynamic (Schuftan, 2015) and nurtured parity of Indigenous and Western knowledges (United Nations Department of Economic and Social Affairs, 2014, 2018).

## Discussion

Ulu (breadfruit) is the traditional Polynesian staple and frequently, used as a metaphor for a new beginning. In project year one, INSPIRE set out to accomplish the most difficult tasks first—establishing the foundation for RCS in culturally rich, yet resource-constrained A.S. Referred to culturally as Fa’apotopoto ulu mai paranesi mamo muamua (gather breadfruit from the farthest branches first). INSPIRE sought to extend Indigenous innovation by weaving Samoan and Western ways of knowing by tailoring communications to promote community access of the resource hub. Findings indicate that INSPIRE’s efforts were feasible and advanced year one aims.

### Limitations

Generalisation of findings on the pilot curriculum is limited by methodologic issues. First, trainees voluntarily applied for participation. Voluntary participation advantages the perspectives of those already interested in research strengthening, SDOH, disparities and/or health equity and who may already identify as change agents. Secondly, there was one individual who did not complete the training due to family-related commitments and did not provide feedback on their experience. Thirdly, trainee and community feedbacks most often were gathered through group discussion facilitated by INSPIRE partners; thus, feedback is subject to social approval bias and advantages those who are more verbal. Lastly, trainees preferred group work and made it more difficult to assess individual learning. These limitations point to implications for future research praxis. In the future, INSPIRE will include more written formative evaluation, as well as summative evaluation that further indicates ‘what’ works in enhancing research knowledge and application.

### Strengths

INSPIRE’s promotion of the research hub as accessible to all reflects Samoan values of collectivism; the value of knowledge as collectively produced and owned is specifically reflected. In the modern idiom, this is operationalised by networks of organisations working together for the common good. INSPIRE’s formalised association with the STEM steering committee and Territorial Epidemiological Outcome Workgroup offers the opportunity for resource-sharing and creation of cross-cutting research. Cooperation with STEM extends INSPIRE’s reach to students who may be positively exposed to social and public health sciences, thereby potentiating a pipeline for future researchers and a source of sustainable research capacity.

Although the impact of each training session on trainee knowledge and attitudinal change was not assessed by written evaluation this was offset at least partially, by weekly group discussions/de-briefings with INSPIRE partners, trainees and regularly scheduled community advisory board meetings. Trainees’ knowledge and skill levels are suggested by the voluntary participation of four trainees in development of this article and in their initiation of Le Fale o So’ofa’atasiga model for RCS in A.S. These learning deliverables suggest that participating trainees understood curriculum content and were able to apply weaving of Indigenous and Western knowledges and culturally tailoring approaches.

Partnership efforts were strengthened by a history of positive collaboration on previously conducted research activities, including the application that currently funds INSPIRE. All partners entered the initial project phase with a shared commitment to action-oriented and community-based participatory research approaches, as well as intent to lalaga (weave) Western and Samoan knowledges. Through previous collaboration, partners had established protocols and processes for engagement and agreements on local ownership, routine updates and respectful communications (Ka‘opua *et al.*, 2011b, 2014; Essence on Health Research, 2014). A.S. partners are purposefully designated as the principal investigators and the final arbiters of programme decisions. INSPIRE partners commit to nurturing a relational climate of cultural safety and humility, with respect for all ways of knowing and conscious subversion of unequal power in collaboration. In short, INSPIRE partners are conscious of the ‘West lectures the rest dynamic’ and seek to view difference through the lens of cultural humility (Schuftan, 2015; Cultural Survival, 2018; United Nations Division for Sustainable Development, 2018).

A test of cultural humility occurred when A.S. investigators observed ‘le fa’alavelave’ (‘the interruption in everyday life’), a term commonly used to describe customary family and community rituals associated with the passing of a loved one) (Milner, 1993). Initially, US co-investigators were unfamiliar with the time-intensive nature of the custom and wondered ‘how’ le fa’alavelave would influence the delivery of time-sensitive deliverables. The US co-investigators engaged in critical reflection and came to realise that fa’alavelave was an opportunity to practice cultural humility, support the A.S. partners in their loss, examine our personal values and appreciate fa’alavelave as the cultural means by which le aiga potopoto (family, community) share love and solidarity in times of grief.

INSPIRE was guided by the RCS best practices literature to purposefully assure local ownership, strong support, supervision and mentorship structures (Essence on Health Research, 2014). Twinning was a key strategy used and involved frequent pairing of A.S. principal investigators with US co-investigators. This strategy was effective in development of funding applications, training curriculum and manuscripts prepared for peer-reviewed presentations and publication. Twinning on INSPIRE activities potentiated mutuality in learning.

### New ways of thinking about SDOH, democratic deficiencies, cultural survivance

Our work brought us to study more comprehensively SDOH in A.S. In our comparison of political, socioeconomic and data challenges, we found striking differences between the US states and the five US territories. INSPIRE’s comparison of the US territories with the US states, alerts us to the many ways in which territorial status operates as a pernicious SDOH. However, we would be remiss if we did not present a perspective on Samoan survivance—a view that seems prevalent in A.S. According to Tapa’au (Chief) Dr Daniel Aga, democratic deficiencies exist in external relations with the USA as plenary power (UN, 2017). However, cultural sovereignty and survivance exist in A.S.’ internal relations (i.e. relations within the A.S. homeland). Cultural sovereignty ensures the perpetuation of Samoan language, traditional village governance by matai (chiefs) and land tenure rights tied to kinship and village systems. In other words, land use remains under the stewardship of Samoan people. In the contemporary idiom, Samoans may purchase land for non-commercial use, pass title to heirs and sell land to other Samoans, thereby safeguarding Indigenous land tenure and stewardship. From this Indigenous perspective, Samoans are neither colonised nor victims. Fa’asamoa (Samoan ways) with its concomitant cultural–linguistic traditions remain part of everyday life; Samoans make decisions on local community issues and cultural sovereignty. Such cultural sovereignty allows for the perpetuation of a way of life that nourishes Indigenous ways of knowing and offers a legacy that may be passed on to future generations (Vizenor, 2008).

### Lessons learned, considerations for discussions on sensitive issues

When seeking to address SDOH with Indigenous communities, it is crucial for non-Indigenous partners to engage in discussions on Indigenous perceptions of colonisation, cultural sovereignty and Indigenous survivance. This may involve discussions protracted across time, as the subject matter is multi-faceted. Use of discussion practices grounded in the culture of the Indigenous people may enhance creation of a culturally safe environment. INSPIRE partners from the US states sought to better understand issues related to systemic discrimination, cultural sovereignty and survivance from the Samoan perspective. Social workers co-facilitated discussions between US and A.S. partners. US partners learned that treading the sensitive balance of addressing SDOH in A.S. involves cultural humility specifically, the willingness to learn deeply and respect authentically perceptions of colonisation, survivance and sovereignty that may be considerably different from a Westernised social work perspective. To ward off cultural–political evangelism, a variant of the ‘West lectures the rest’ dynamic, INSPIRE’s US-based researchers learned to be mindful of pre-conceived biases and were willing to struggle towards new understanding (Schuftan, 2015; Cultural Survival, 2018). Such understanding is vital to weaving diverse, yet complementary research knowledges and use of culturally tailored communication strategies. An Indigenous elder reminds us that ‘deep cultural understanding gives substance to form’.

## Conclusion

The call to action on the SDGs (United Nations Division for Sustainable Development 2018) is to ‘leave no one behind and reach the furthest first.’ This call aligns with the values of social work and other helping professions. Through this lens the SDG agenda may be viewed as an opportunity for inter-professional collaboration with local and global communities affected by disparate health outcomes and deleterious SDOH (Jayasooria, 2016). Indigenous communities and SIDS historically, have been left behind and continue to face unique challenges in addressing SDOH (United Nations Department of Economic and Social Affairs, 2014, 2018). Unique challenges to A.S. as a US territory are descibed in an earlier section and suggest that territorial status may function as a deleterious, distal SDOH. This observation may be extended in future research.

Jayasooria (2016) advocates social work’s participation and service leadership at the local and global levels but offers critical anticipatory guidance. Sociocultural and political controversy may arise. Social workers and their partners likely will be challenged to critically reflect and discuss sensitive matters such as: What history informs our perspectives? Whose ways of knowing matter in knowledge-building? What languages and communication medium are given preference in knowledge development and dissermination? These and other similar questions may surface repeatedly and prompt examination and ongoing dialogue on issues that hinder partnerships from realising their full potentional (Ife, 2001; Jayasooria, 2016). In this article, we describe examples of controversial issues and the vitally important, *albeit* uncomfortable discussions that ensued. Through engaging in discussion with cultural humilty, INSPIRE partners moved to deeper levels of understanding and affirmed mutual commitment to service leadership aimed at addressing pernicious, seemingly intractable SDOH, promoting health equity and ensuring local ownership. This supports the cultural belief of O le ala i le pule o le tautua (the pathway to leadership is through service to others). Such discussions may be viewed as the weaving of diverse perspectives and are opportunities for fostering health innovations and community ownership. As emphasised by the International Federation of Social Work (Truell and Jones, 2012), sustainable global health and health equity may only be achieved when human relationships also are sustained.
